# Injection Compression Molded Microlens Arrays for Hyperspectral Imaging

**DOI:** 10.3390/mi9070355

**Published:** 2018-07-18

**Authors:** Marcel Roeder, Marc Drexler, Thilo Rothermel, Thomas Meissner, Thomas Guenther, André Zimmermann

**Affiliations:** 1 Hahn-Schickard, Allmandring 9B, 70569 Stuttgart, Germany; Marc.drexler@hahn-schickard.de (M.D.); Thilo.rothermel@hahn-schickard.de (T.R.); Thomas.meissner@balluff.de (T.M.); Thomas.guenther@hahn-schickard.de (T.G.); zimmermann@ifm.uni-stuttgart.de (A.Z.); 2 Institute for Micro Integration (IFM), University of Stuttgart, Allmandring 9B, 70569 Stuttgart, Germany; 3 Balluff GmbH, Schurwaldstraße 9, 73765 Neuhausen auf den Fildern, Germany

**Keywords:** microlens array, ultraprecision milling, injection compression molding, microstructures, polymer optics, hyperspectral imaging

## Abstract

In this work, a polymer microlens array (MLA) for a hyperspectral imaging (HSI) system is produced by means of ultraprecision milling (UP-milling) and injection compression molding. Due to the large number of over 12,000 microlenses on less than 2 cm², the fabrication process is challenging and requires full process control. The study evaluates the process chain and optimizes the single process steps to achieve high quality polymer MLAs. Furthermore, design elements like mounting features are included to facilitate the integration into the final HSI system. The mold insert was produced using ultraprecision milling with a diamond cutting tool. The machining time was optimized to avoid temperature drifts and enable high accuracy. Therefore, single immersions of the diamond tool at a defined angle was used to fabricate each microlens. The MLAs were replicated using injection compression molding. For this process, an injection compression molding tool with moveable frame plate was designed and fabricated. The structured mold insert was used to generate the compression movement, resulting in a homogeneous pressure distribution. The characterization of the MLAs showed high form accuracy of the microlenses and the mounting features. The functionality of the molded optical part could be demonstrated in an HIS system by focusing light spectrums onto a CCD image sensor.

## 1. Introduction

The applications for polymer optics are growing significantly in recent years. Advantages in the fabrication process and improvements in the material’s properties enable polymer optics to compete with traditional glass lenses. Applications for polymer optics can be found in the fields of medical engineering, automotive, illumination, sensors and measurement systems. Especially the comparably easy and fast fabrication of freeform and micro structured optics are significant advantages compared to traditional glass lenses. Therefore, limitations in the available refraction indices of transparent polymer materials can be compensated by advanced form and optical design. Examples for microstructured optics are Fresnel lenses [1], microprism arrays [2], diffractive optical elements [3], and microlens arrays (MLA) [4].

In this work, a polymer MLA is fabricated. MLAs can be used in color imaging [5], fingerprint identification [6], 3D light field cameras [7] and hyperspectral imaging (HSI) [8]. The fabrication of MLAs can be performed with a multitude of methods. Examples are ultraprecision machining [9], LIGA (Lithographie Galvanik Abformung) [10], E-beam writing [11], laser machining [12], and electric discharge machining [13]. The biggest challenge for most of the fabrication methods is to achieve optical surface quality with Ra < 10 nm. With E-Beam writing and laser machining, it is possible to create surfaces with surface roughness of about Ra = 10 nm and higher [14]. Sub-10 nm surface roughness remains challenging. The LIGA process however is able to achieve surface qualities of Ra < 10 nm, similar to the ultraprecision machining process [9,10]. The resulting surface quality is also strongly depending on the substrate material which is a limitation of all technologies. When high resolution and surface quality is needed, the machining has to be performed on soft or brittle materials which cannot be used in injection molding. Therefore, a subsequent electroplating process is mandatory to create a solid mold insert. An advantage of these technologies is the machining time which can be very fast, depending on size and shape of the MLA. The fabrication of MLA mold inserts by means of electric discharge machining is still in an early stage, so further improvements are necessary for the technology to compete with the other methods. Especially the resulting surface roughness with > 100 nm is not suitable for optical applications [15].

For this work, the MLA is fabricated using ultraprecision milling (UP-milling) and injection compression molding. The UP-milling is used to produce an optical mold insert that can be transferred into a molding tool. Therefore, a diamond milling tool with a defined cutting edge is used. UP-milling is broadly used for the fabrication of optical mold inserts, especially freeform optics [16] and MLAs [17]. The main advantages of the technology are the resulting optical surface quality with Ra < 10 nm without necessity of a post-treatment as well as very high form accuracy in the micrometer range [18]. However, diamond machining must be performed on non-ferrous materials when no special machining equipment like ultrasonic machining is available. Otherwise, increased tool wear will significantly affect the resulting surface quality and form accuracy. Therefore, nickel–phosphorous (NiP) coatings are usually applied for the fabrication of ultraprecision machined mold insert.

Injection molding and hot embossing [19] are commonly used technologies for the replication of polymer optics. Especially injection compression molding is used, when high quality polymer optical components are required [20]. Injection compression molding is a process variant of regular injection molding. Thereby, a compression stamper is used to generate a homogeneous pressure distribution during the molding process. Thus, homogeneous material density within the polymer part is achieved, resulting in components with low birefringence. Furthermore, improved form accuracy is possible. A detailed description of the process can be found in the literature [21].

The MLA produced in this work is applied in a HSI system. Thereby each individual microlens focuses a light spectrum on a photo sensor. HSI has many applications in the fields of medicine [22], agriculture [23], astronomy [24] and food processing [25]. Due to the fact, that a spectrum is obtained for every microlens, detailed information on the investigated object can be acquired with spatial information.

The aim of this paper is to demonstrate a successful process chain for the fabrication of polymer MLAs with focus on the mold design, mold insert fabrication by means of UP-milling and replication by injection compression molding. In the first section of the paper the design of the MLA and the molding tool are described as well as the fabrication methods. Subsequently, the results of the ultraprecision milling and the injection compression molding are shown. In the last section of the paper, the results are discussed and reviewed.

## 2. Materials and Methods 

### 2.1. Microlens Array and Mold Design

**Figure 1:**
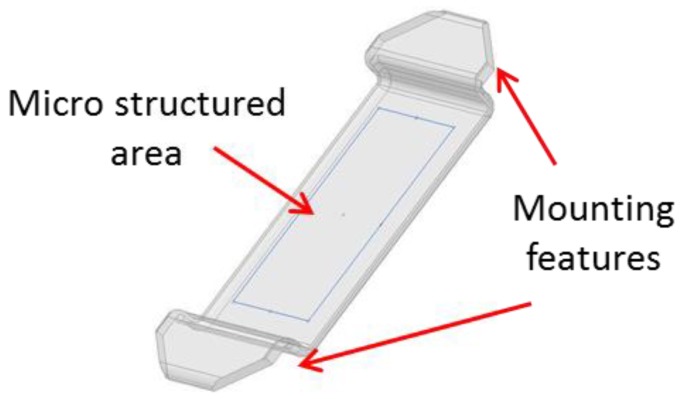
Design of the polymer MLA with the microstructured area and mounting features.

The MLA fabricated in this work comprises of a structured area of 13 × 5 mm. Every individual microlens has a radius of 1 mm and a spherical shape. The pitch between the microlenses is 127.5 µm, resulting in a total of about 12,000 lenses for the whole MLA. On the polymer MLA, the microlenses are convex with a sag height of 4 µm. Furthermore, the MLA includes mounting features to enable the integration into a HSI system. The microlenses have to be orientated in an angle of 26.56° to focus the light onto the pixels of a photo sensor. The designed part thickness of the polymer MLA is 500 µm. The design of the MLA is shown in Figure 1.

For the replication process, an injection molding tool is required. The molding tool is designed as an injection compression molding tool for main axis compression. For the injection compression molding process the molding tool is equipped with a moveable frame plate. The frame plate can be moved separately from the rest of the molding tool using pneumatic cylinders. Before every molding process, the frame plate is moved into a front position to close the cavity before melt injection. Thus, a compression movement can be performed after the material is injected into the cavity. This mold setup can generate high compression forces even on electrical molding machines. The molding tool includes two mold inserts, each on one side of the molding tool, which form the shape of the polymer MLA. The mold insert on the fixed side represents the rear part of the MLA, consisting of a flat optical surface. On the moveable side, the mold insert with MLA structure is included. This mold insert is used as the moveable compression stamper during the molding process. More details on the fabrication of the mold inserts are provided in the following sections. The molding tool consists of a single cavity with a runner system through the center of the fixed side.

**Figure 2:**
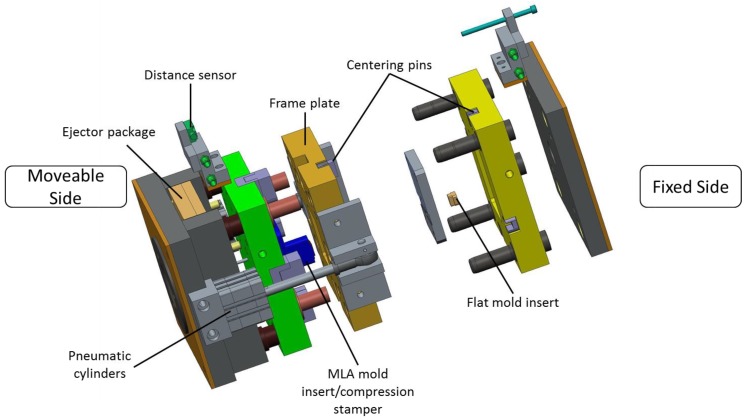
CAD drawing of the injection compression molding tool in an exploded view.

Additionally, the molding tool is equipped with a distance sensor, fixed onto the molding tool, which is connected to the molding machine. In this way, accurate positioning of the molding tool and compression stamper is possible which is mandatory to achieve accurate and repeatable results. The distance sensor allows a more accurate positioning compared to the internal measurement system of the molding machine. Accurate positioning of the mold parts is a key element when injection compression molding is employed. The molding tool has four separate cooling circuits which are controlled by individual tempering systems. Each mold side is tempered individually by external water tempering systems. Furthermore, the compression stamper and the distance sensor are equipped with separate tempering systems. Thus, precise temperature control of the individual components is possible. A CAD drawing of the molding tool is shown in Figure 2.

### 2.2. Mold Insert Fabrication

The fabrication of the mold inserts is one of the most crucial steps in the fabrication of the polymer MLAs. Inaccuracies and form deviations cannot be compensated in the replication process. The mold inserts are fabricated using a five-axis ultraprecision machine (Freeform 700a, AMETEK Precitech Inc., Keene, NH, USA). The machine is equipped with hydrostatic bearing axes, temperature control, and an air bearing spindle. Due to the fact, that ferrous materials cannot be machined by diamond cutting tools, the steel mold inserts are coated with a nickel–phosphorous coating (SuNiCoat^®^ Optics, CZL Tilburg, Tilburg, The Netherlands). After the coating, the mold insert is premachined by means of standard milling and grinding to remove spared material. Afterwards, a fly-cutting process is used to create a plane surface on the mold inserts with optical quality. For the mold insert on the fixed mold side, this is the final processing step. For the microstructured mold insert, the fly-cutting process is mandatory to create a flat and even surface for the subsequent UP-milling process. The fly-cutting tool has a diameter of 25 mm and a diamond with a 3-mm cutting edge (Matzdorf GmbH, Nürnberg, Germany). The spindle speed was set to 1500 1/min at a feed rate of 20 mm/min. Prior to the machining process, the fly-cutting tool was set to an exact 90° angle to the mold insert surface. All machining processes on the ultraprecision machine are performed using isoparaffin as a cooling lubrication sprayed on the machining area and a controlled temperature in the machining chamber of 22 °C ± 0.1 °C.

**Figure 3:**
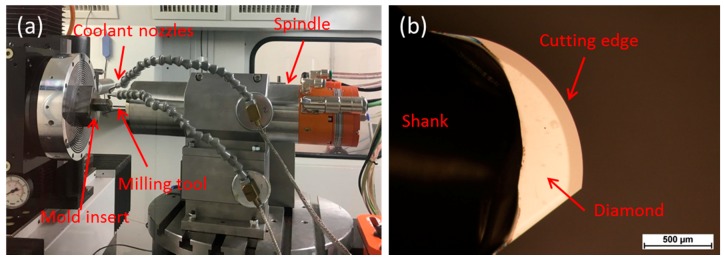
(**a**) UP-milling of the mold insert; (**b**) Cutting edge of the diamond cutting tool.

The MLA microstructured mold insert is fabricated using UP-milling. Due to the large number of microlenses that have to be machined, machining time is a significant quality factor to avoid temperature drifts. Thus, a milling strategy with minimal machining time for each microlens has to be chosen. Therefore, a single immersion of the diamond milling tool is applied. A detailed description of the milling process is published elsewhere [26]. Using this strategy, the quality of the milling tool is the most important factor, since the shape of the diamond exactly represents the resulting microlens shape. The milling tool used in this work had a radius of 0.9993 mm with a waviness < 50 nm, measured by the manufacturer. The milling tool was provided by Contour Fine Tooling (Contour Fine Tooling BV, Valkenswaard, The Netherlands). A picture of the milling process as well as the diamond cutting tool is shown in Figure 3.

For the UP-milling process of the MLA mold insert, the spindle speed was set to 70,000 min^−1^. Prior to the milling process, the milling spindle had a lead time of 10 min to reach the operating temperature of the system. Otherwise, spindle elongation due to internal heating will affect the milling process. Although the sag depth of each microlens is only 4 µm in the mold inset, the diamond tool was immersed 9 µm into the material to compensate potential unevenness on the substrate surface. The feed rate during the tool immersion was set to 2.5 m/min, during the repositioning of the milling tool the feed rate was increased to 50 mm/min to reduce processing time. The milling tool was immersed in a 20° angle to the substrate surface to compensate potential decentering of the diamond on the tool. The machining parameters used for the fabrication of the MLA mold insert are shown in Table 1.

### 2.3. Injection Compression Molding

**Figure 4:**
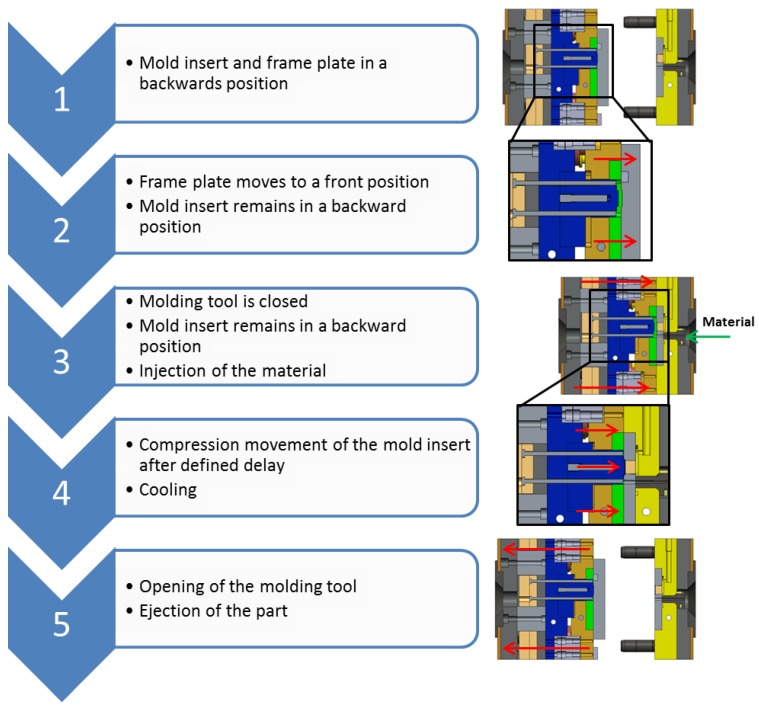
Process steps of the injection compression molding process.

The injection compression molding is performed on an Arburg Allrounder 370A molding machine (Arburg GmbH & Co. KG, Loßburg, Germany) with vertical melt injection unit. As a molding material, Zeonex COP 330R (Zeon Cooperation, Tokyo, Japan) was used due to its low autofluorescence and good optical properties. The injection compression molding process performed for the MLA fabrication can be divided into five process steps, which are shown in Figure 4. At the beginning of the process, the structured mold insert and frame plate are in a backwards position. Then, the frame plate of the molding tool is moved into the front position by means of pneumatic cylinders. Afterwards, the molding tool is closed. Thereby, the frame plate creates a sealed cavity even though the structured mold insert remains in a defined backward position. This is important to ensure the complete filling of the cavity. If the mold insert is moved to the front to early, the resulting gap is too small, preventing the melt to completely fill the cavity due to high flow resistance. The third process step is the injection of the melt into the cavity. With a defined delay, the compression movement with the structured mold insert is started, moving the insert to a defined position. Thus, a homogeneous pressure within the cavity is created. In the last step of the process, the molding tool is opened and the resulting MLA ejected. The most crucial aspects in the process are the positioning of the structured mold insert prior to injection, delay of the compression movement and final position of the structured mold insert. The interplay of these parameters has to be defined carefully to obtain high quality optical components.

The molding parameters used for the replication of the MLAs are shown in Table 2.

## 3. Results

### 3.1. Mold Insert Fabrication

**Figure 5:**
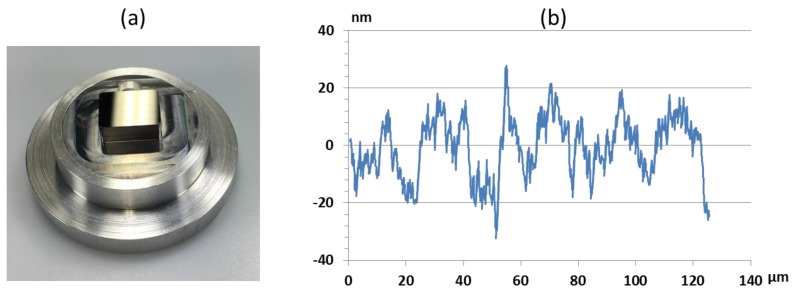
(**a**) Mold insert with flat optical surface after fly-cutting process; (**b**) Surface profile acquired by WLI.

Two fly-cutting processes were performed on the mold inserts for the fixed and moveable mold side. At first, the mold insert for the fixed side was machined. The machined area of the mold insert is 10 × 13 mm. Due to the fact, that the diameter of the fly-cutting tool is larger than the mold insert, a single cutting transit is sufficient to machine the area. The resulting surface represents the backside of the MLA. Therefore, the quality of the surface is important for the optical performance of the resulting polymer MLA. For the cutting process, a cutting depth of 3 µm was chosen. The resulting surface roughness was Ra = 12 nm, measured by white light interferometry (WLI, WYKO NT9100a, Veeco Instruments Inc., Plainview, NY, USA). The mold insert and the surface measurement are shown in Figure 5.

**Figure 6:**
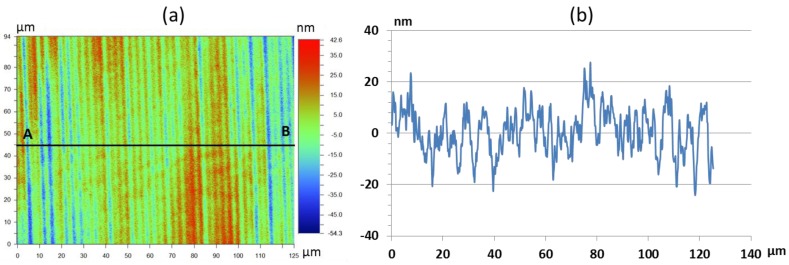
(**a**) WLI Surface measurement of the mold insert after the fly-cutting process, (**b**) extracted 2D-profile along AB.

The second fly-cutting process was performed to create a flat surface for the subsequent UP-milling process. The same cutting conditions as applied in the previous fly-cutting process were used. The resulting surface roughness was similar to the prior process with Ra = 12 nm. The measurement results are shown in Figure 6.

**Figure 7:**
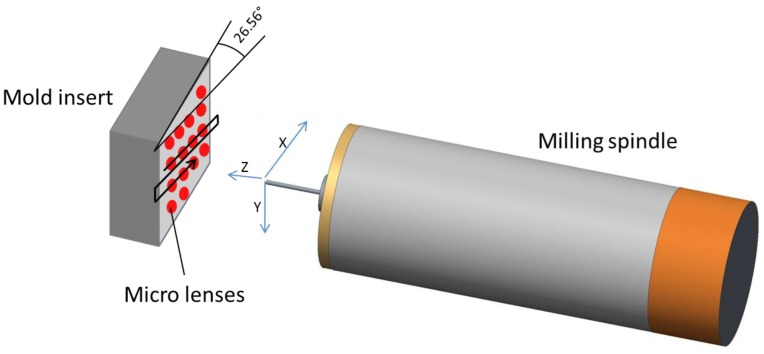
Milling strategy for the fabrication of the microlenses.

For the fabrication of the microlenses machined into the mold insert, an UP-milling process was used. The mold insert was positioned in a defined angle of 26.56°. Thus, the machining of the individual microlenses could be done in a way that they are oriented in a horizontal line. This is an advantage during the milling process, since only two axes have to be moved (*x*-axis and *z*-axis), resulting in reduced positioning errors. The third axis (*y*-axis) only has to be moved when another line of microlenses has to be started. The milling strategy is illustrated in Figure 7. The milling time for an individual lens was about 1 s. The resulting process time for the whole mold insert was about 4.5 h, since the repositioning after every horizontal line adds to the machining time.

**Figure 8:**
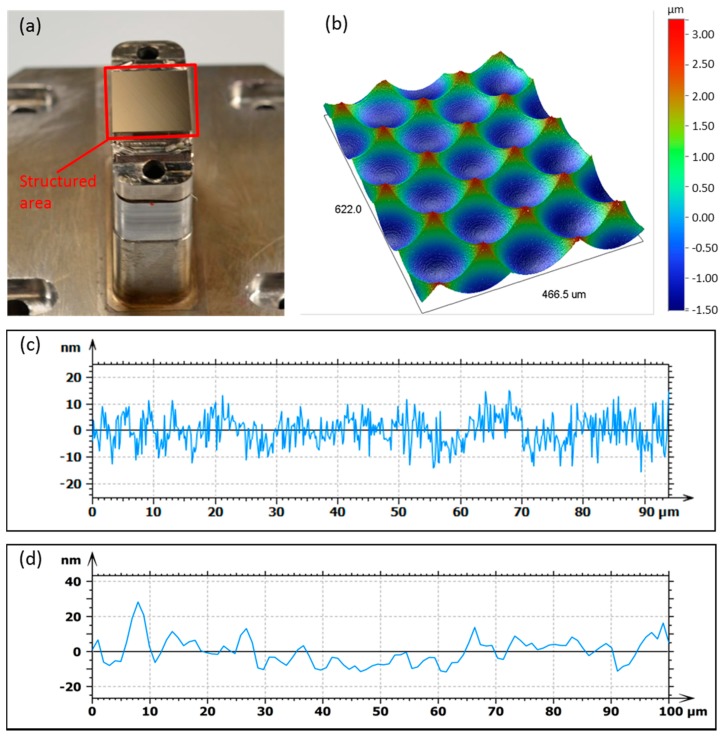
(**a**) Mold insert after UP-milling; (**b**) WLI measurement of the MLA on the mold insert; (**c**) Surface roughness of a single microlens with Ra = 4 nm, (**d**) Form deviation of a single microlens from a 1-mm radius measured with Mitaka MLP-3.

The resulting MLA mold insert is shown in Figure 8. The surface roughness of a single microlens was Ra = 4 nm, measured by WLI. The roughness depth was Rt = 27 nm. For the evaluation of the surface roughness the overall curvature was mathematically removed. Furthermore the form deviation of the microlenses was analyzed using a laser autofocus probe measurement system (Mitaka MLP-3, Tokyo, Japan). The resulting form deviation of P-V 39.5 nm from the required 1 mm radius is shown in Figure 8d. The distance between the microlenses of 127.5 µm, which equals the diameter, could be achieved very accurately and no drift in the distance could be measured.

### 3.2. Injection Compression Molding

**Figure 9:**
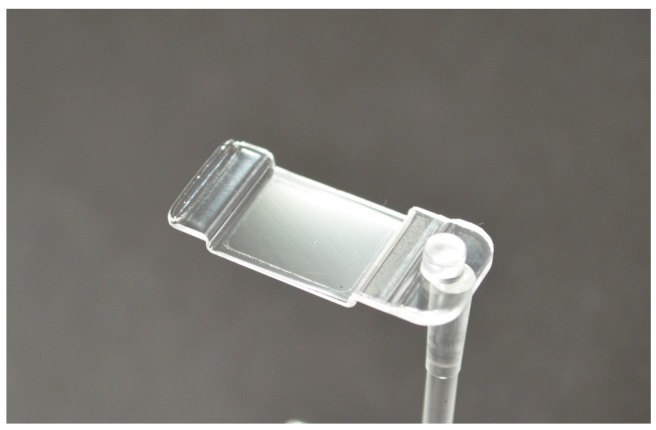
Injection compression molded MLA before the sprue is removed.

The molding process was performed by starting with a regular injection molding process to evaluate the process parameters regarding filling behavior in the cavity. Accordingly, no compression movement was carried out. Afterwards, the compression movement was included to improve the optical quality of the components. This approach proved to be suitable to establish injection compression molding processes, since the quality of the parts is affected by a multitude of factors. Splitting the process into two steps, simplifies the optimization of the molding parameters. Molding parameters were adjusted in a way to improve form accuracy and filling behavior. Cycle time was not a relevant factor in this work. One of the most critical aspects of this molding process was the coordination of the moveable structured mold insert with the injection of the material. Due to the limitation of the part thickness to 500 µm, material flow is limited and the material cooling occurs rapidly. The result was a narrow process window in which a sufficient part quality could be obtained. The process steps of the compression molding process are described in Section 2.3 (Figure 4). Before the injection phase, the structured mold insert is positioned in a backward position, about 0.9 mm from the end position. Thus, at the beginning of the material injection there is a larger cavity allowing a faster filling of the cavity. With 0.7 s delay, the compression movement is started. At this point, the injection phase is already completed, since the material injection only takes 0.8 s. In contrast to the part thickness, the replication of the micro structured area is not a limiting factor. Since the microlenses are relatively flat with no significant aspect ratio, the filling of the microlenses structures does not represent a challenge and in combination with the application of a compression force, the micro structures are replicated accurately without necessity of adjusting the molding parameters. An injection compression molded MLA with sprue is shown in Figure 9. For the integration of the MLA into the HSI system, the sprue has to be removed mechanically.

**Figure 10:**
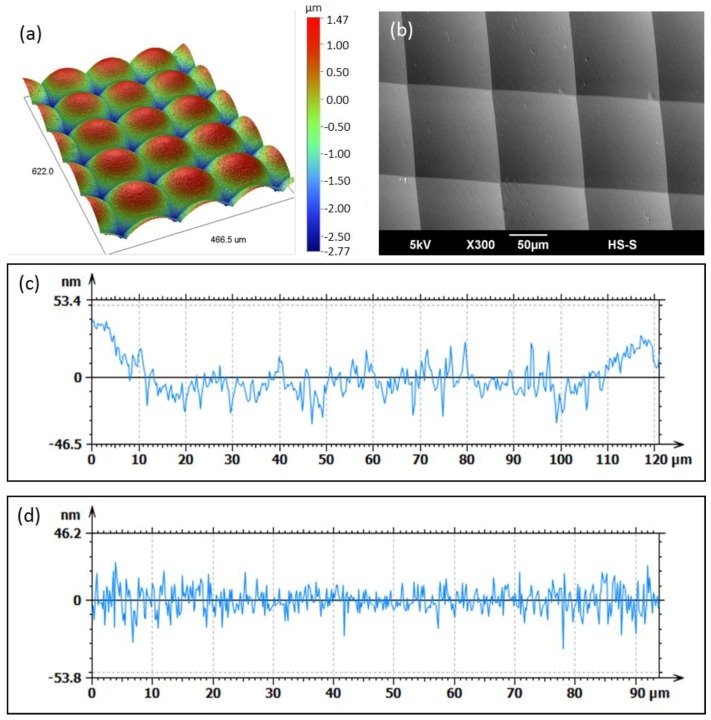
(**a**) WLI measurement of the molded microlenses; (**b**) SEM image of the molded MLA; (**c**) Form deviation from a 1 mm radius measured with Mitaka MLP-3; (**d**) Roughness measurement by WLI with Ra = 6 nm.

The WLI measurements of the micro structures show a homogeneous distribution of the microlenses with no visible deviations (Figure 10a). A single microlens of the molded MLA was characterized using the Mitaka MLP-3 system (Figure 10b). The measurement shows, that the sag height of about 4 µm is fully replicated. The form deviation from the aimed 1 = mm radius is about P-V 80 nm for a single lens (Figure 10c). The surface roughness of a single microlens was measured to be Ra = 6 nm. However, the measurement of the roughness depth resulted in Rt = 53 nm. Both measurements were performed using WLI. For the evaluation of the surface roughness the overall lens radius was mathematically removed.

**Figure 11:**
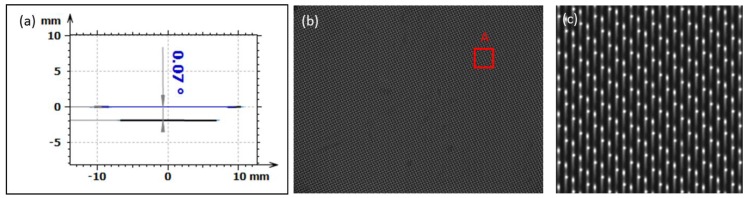
(**a**) Angle of 0.07° between the mounting features and the microstructured area measured with laser autofocus probe system; (**b**) Image of the resulting light spectra on the camera system using the molded MLA; (**c**) Close view of the light spectra at position A.

Since the MLA needs to be integrated into a hyperspectral imaging system, the planarity between the mounting features on the sides and the micro structured area is an important factor for the application. Using a laser autofocus probe measurement, the angle between the mounting features and the structured area was measured to be 0.07° (Figure 11a). To show the functionality of molded MLA, the part was integrated into a hyperspectral imaging system. The resulting image on the camera chip is shown in Figure 11b. The light spectra generated by the hyperspectral imaging system are homogeneously distributed throughout the whole area, showing that the molded MLA performs as required. A detailed characterization of the hyperspectral imaging system is not part of this work and will be published elsewhere.

## 4. Discussion

The results presented in this work show that to combination of ultraprecision machining and injection compression molding is a suitable process chain to fabricate polymer MLAs with a high number of microlenses. In the following, the results as well as each process step is critically reviewed and discussed.

Fly-cutting proved to be a suitable method to prepare the optical mold inserts. It combines fast machining with low surface roughness. The resulting surface roughness of Ra = 12 nm corresponds to values found in the literature [27]. Particularly, since the mold inserts are not rotationally symmetric, fly-cutting is a more suitable method to create a flat surface then ultraprecision diamond turning. However, accurate positioning of the fly-cutting tool and the mold insert are a key to obtain an optical surface quality.

**Figure 12:**
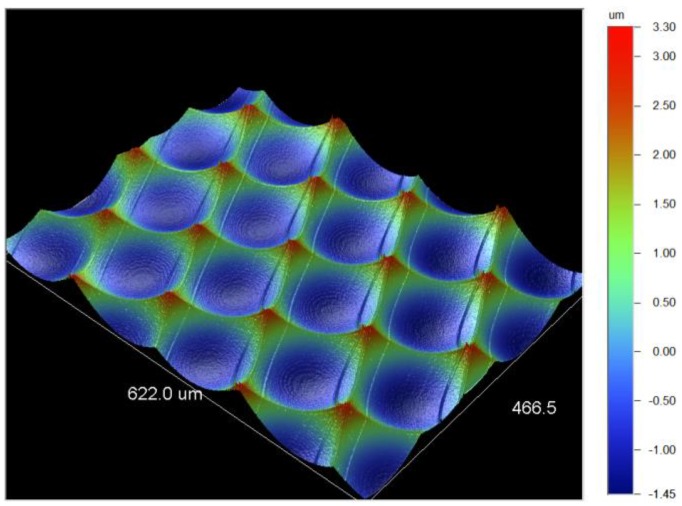
MLA on a test substrate with resulting microlenses after significant tool wear of the cutting diamond measured by WLI.

The milling strategy that was chosen for the fabrication of the MLA mold insert proved to be suitable to create accurate microlenses with homogeneous dimensions. Due to a single immersion of the milling tool, machining time could be kept at 4.5 h for 12,000 microlenses. However, the applied milling strategy also comes with several restrictions, which limits the range of application. The most important factor for the process is the quality of the milling tool. The radius of the cutting diamond, waviness and positioning of the cutting diamond need to be very accurate in the submicrometer range. Notably, the milling strategy can only be applied when the MLA consists of microlenses with the same radius. Furthermore, microlenses with aspheric or free-form shape are not possible. In these cases, different milling strategies like spiral immersion are necessary. Changes in the radius of the microlenses and the pitch can be easily adjusted with the proposed strategy. A critical factor in the machining process is tool wear, since defects on the diamond cutting edge will inevitably result in marks in the microlenses. An example of a MLA after milling with a worn cutting diamond is shown in Figure 12.

Tool wear can be reduced when the cutting depth is kept at a minimum and cooling lubricant is used. Nevertheless, tool wear remains a factor which is difficult to predict. Due to the fact that the individual microlenses need to be positioned very accurately, exchanging the diamond cutting tool during the machining process is no valid option. Recalibration of a new cutting tool cannot be performed with the required accuracy.

Compared to other applicable fabrication technologies like e-beam writing and laser machining, the ultraprecision machining process achieves higher surface quality, comparable to what can be obtained with the LIGA process. Furthermore, the mold insert could be machined directly without any further post-treatment or electroplating process. Compared to the other technologies, machining time is in the same range [12,28]. Laser machining can produce faster and more microlenses, when the lenses are very small (diameter < 10 µm) [14].

The resulting MLA mold insert fulfilled all the required properties considering form accuracy of the microlenses, surface roughness, and positioning of the microlenses. The resulting form deviation of < 40 nm in the mold insert is very low and could only be achieved due to the high quality and form accuracy of the diamond cutting tool. The resulting surface roughness of an individual microlens of Ra = 4 nm in the mold insert fulfills the requirements for optical applications. It is important to mention that the measurement of the microlens radius does not generate reliable results due to the fact that only a small section of the 1-mm radius is available for the measurement. Small deviations and surface roughness affect the calculation of the resulting radius, leading to high inaccuracy. Therefore, it is advisable to analyze the form deviation from the required radius instead of only measuring the radius.

The molded MLA was fabricated by injection compression molding. Due to the required part thickness of 500 µm, the process window was significantly limited. The complete filling of the cavity needed to be fast to avoid the cooling of the material before the cavity was filled. To improve the filling behavior, the cavity was increased during the material injection by keeping the structured mold insert in a backwards position. Due to this strategy, an injection compression molding tool was mandatory to allow the additional movement of the molding tool. Keeping the structured mold insert in a backwards position enabled a fast injection of material and, subsequently, the part could be compressed to the required part thickness. However, the movements of the structured mold insert which is used as a compression stamper is critical and needs to be timed accurately with the injection phase to obtain accurate molded parts. A regular injection molding process was also tested, but entire parts could not be obtained. The resulting surface roughness of Ra = 6 nm complies with the required optical surface quality which is needed for the application. Equally, the measured form deviation of ±40 nm is very good and sufficient for the application in the hyperspectral imaging system. Form deviation doubled compared to the mold insert, however, form deviations in the sub-micrometer range are extremely good. The increased form deviation is most likely a result of shrinkage and warpage during the molding process. Since mounting features are included in the molded part, the angle between these features and the micro structured area is critical. A large angle results in a defocus of the image on the camera chip in the hyperspectral imaging system and, therefore, reduces the resolution of the system. The measured 0.07° however does not affect the quality of the system.

## 5. Conclusions

To summarize the results, it can be concluded that the combination of ultraprecision milling and injection compression molding is a suitable process chain for the fabrication of high quality molded MLAs. The MLA fabricated in this work contains over 12,000 individual microlenses. An ultraprecision milling strategy, where the diamond cutting tool is immersed into the material without any further movement, was determined to be the most suitable method for the fabrication of the MLA mold insert. Thereby, accuracy of the microlenses was the focus, as well as short machining time. Due to the milling strategy, the milling tool is the most crucial factor regarding the quality of the mold insert, since it exactly represents the resulting shape of the microlenses. For the replication of the MLAs, injection compression molding was determined to be a suitable method. Due to the fact that mounting features were included at the MLA and the part thickness was only 500 µm, a customized injection compression molding process was mandatory. To obtain complete filling of the cavity, the structured mold insert needed to be in a backwards position prior to the injection phase, otherwise the required dimensions could not be reached. In summary, it can be concluded that an understanding of the fabrication processes—as well as a high-control ultraprecision machining and injection compression molding—are required to achieve high quality MLAs.

## Figures and Tables

**Table 1:** Machining parameters for the fabrication of the MLA mold insert by means of UP-milling.

Parameter		Value
Spindle speed (min^−1^)		70,000
Tool immersion (µm)		9
Feed rate (mm/min)	Immersion	2.5
	Tool positioning	50
Tool angle (°)		20
Temperature (°C)		22
Tool radius (mm)		0.9993

**Table 2:** Molding parameters.

Parameter		Value
Melt temperature (°C)		235/250/265/265/255/65
Mold temperature (°C)	Fixed side	120
	Moveable side	120
	Compression stamper	120
Holding pressure (bar)		300
Injection pressure (bar)		1250–1400
Injection time (s)		0.08
Cycle time (s)		28
